# The effect of various factors on the dental arch relationship in non-syndromic unilateral cleft lip and palate children assessed by new approach: a retrospective study

**DOI:** 10.1186/s12887-017-0870-4

**Published:** 2017-05-06

**Authors:** Sanjida Haque, Mohammad Khursheed Alam, Mohd Fadhli Khamis

**Affiliations:** 1 Orthodontic Unit, School of Dental Science, Universiti Sains Malaysia, Kota Bharu, Kelantan Malaysia; 2 Orthodontic Department, College of Dentistry, Al Jouf University, Sakaka, Kingdom of Saudi Arabia; 3 Forensic Dentistry/Oral Biology Unit, School of Dental Science, Universiti Sains Malaysia, Kota Bharu, Kelantan Malaysia

**Keywords:** Unilateral cleft lip and palate, Dental arch relationship, EUROCRAN index, Congenital factor, Postnatal treatment factor

## Abstract

**Background:**

Cleft lip and palate (CLP) is one of the most common birth defects. Multiple factors are believed to be responsible for an unfavorable dental arch relationship in CLP. Facial growth (maxillary) retardation, which results in class III malocclusion, is the primary challenge that CLP patients face. Phenotype factors and postnatal treatment factors influence treatment outcomes in unilateral cleft lip and palate (UCLP) children, which has led to a great diversity in protocols and surgical techniques by various cleft groups worldwide. The aim of this study was to illustrate the dental arch relationship (DAR) and palatal morphology (PM) of UCLP in Bangladeshi children and to explore the various factors that are responsible for poor DAR and PM.

**Methods:**

Dental models of 84 subjects were taken before orthodontic treatment and alveolar bone grafting. The mean age was 7.69 (SD 2.46) years. The DAR and PM were assessed blindly by five raters using the EUROCRAN index (EI). Kappa statistics was used to evaluate the intra- and inter-examiner agreement, chi square was used to assess the associations, and logistic regression analysis was used to explore the responsible factors that affect DAR and PM.

**Results:**

The mean EUROCRAN scores were 2.44 and 1.93 for DAR and PM, respectively. Intra- and inter-examiner agreement was moderate to very good. Using crude and stepwise backward regression analyses, significant associations were found between the modified Millard technique (*P* = 0.047, *P* = 0.034 respectively) of cheiloplasty and unfavorable DAR. Complete UCLP (*P* = 0.017) was also significantly correlated with unfavorable DAR. The PM showed a significant association with the type of cleft, type of cheiloplasty and type of palatoplasty.

**Conclusion:**

This multivariate study determined that the complete type of UCLP and the modified Millard technique of cheiloplasty had significantly unfavorable effects on both the DAR and PM.

## Background

Cleft lip and palate (CLP) is one the most common congenital anomalies present at birth and is caused by the failure of the palatal shelves to fuse during the embryonic stage [1]. Both congenital (genetic) and environmental factors are thought to be responsible for this malformation [2–4].

Maxillary growth retardation is often observed in patients with repaired unilateral cleft lip and palate (UCLP). Most often, the outcome of treatment for children with UCLP can be assessed by the dental arch relationship(DAR) (outcome of maxillary growth) after cheiloplasty and palatoplasty [5, 6]. The timing and techniques of cheiloplasty and palatoplasty have been found to influence the outcome of the treatment of UCLP [7, 8]. Moreover, type of UCLP, side, family history of cleft and class III malocclusion, and auxiliary intervention also influence the treatment outcome. A lack of consideration of factors affecting the outcome of treatment in children with CLP has led to great diversity in protocols and surgical techniques by various cleft groups worldwide [9]**.** As a result, to ensure the success of the treatment, methods need to be based on sound evidence so that a surgeon can modify their timing or techniques if needed [10].

An assessment of the DAR is considered to be the most valuable benchmark of treatment outcome and can provide important information about facial growth, and it is therefore an important indication of the quality of cleft treatment outcome. The EUROCRAN index (EI) was developed to assess the DAR to evaluate not only the surgical outcomes in patients with UCLP but also the degree of malocclusion in both the antero-posterior and vertical dimensions, as well as the palatal form [7]. This index has already been established as standard index for validity and reliability in the assessment of UCLP [11]. The EI is the only index that evaluates both the DAR and palatal morphology (PM) at the same time, whereas other indices, such as the GOSLON Yardstick [12] and the modified Huddart Bodenham for crossbite [13, 14], evaluate only the DAR.

In recent years, a multitude of research on CLP has been conducted worldwide. In Bangladesh, which is a typical developing country, more than 5000 CLP patients are born every year, and the prevalence rate is 3.9 per 1000 live births [15]. CLP patients in Bangladesh lead an extremely difficult life as they cannot earn a living and sometimes are unable to obtain essential surgical repairs or cleft-associated treatment. Currently, CLP patients in Bangladesh are treated by different organizations, such as NGOs and private hospitals. However, according to surveys in the literature, the treatment outcome or end results of these patients are still unknown, making it difficult to assess different types of treatment. The present study evaluates for the first time the treatment outcome of Bangladeshi UCLP patients based on both congenital/phenotype and postnatal treatment factors using a relatively new index. The treatment outcomes of a CLP patient depend on various factors. However, most studies worldwide have evaluated treatment outcomes based on individual factors [7, 16–19]. A very small number of studies has considered various factors together to determine which factor affects the DAR in UCLP children [20, 21].

We have, therefore, paid particular attention to evaluating the treatment outcomes of Bangladeshi UCLP patients based on both phenotype and postnatal treatment factors using the EI. Based on this, the aims of this study were to:

1. Determine the intra- and inter-examiner reliability of EI scoring.
2. Determine the DAR and PM of Bangladeshi UCLP children using the EI.
3. Determine favorable and unfavorable groups based on the DAR.
4. Evaluate the associations between phenotype and postnatal treatment factors with favorable and unfavorable DAR and PM.
5. Explore the associations between individual factors in terms of favorable and unfavorable DAR using crude logistic regression analysis.
6. Explore the responsible factors for favorable and unfavorable DAR using stepwise backward logistic regression analysis.
7. Present the global and present study EI scores.

## Methods

All participants’ parents provide written informed consent prior to the surgeries. This study was approved by the Ethical Committee of the Hospital Universiti Sains Malaysia (HUSM) [USM/JEPem/15020039], which complies with the Declaration of Helsinki. The current study design was retrospective in nature. A chart review was carried out to identify the subjects born with UCLP in a renowned hospital of Bangladesh. The inclusion criteria of our study included the following: non syndromic UCLP subjects with age range from 5 to 12 years, cheiloplasty and palatoplasty had been performed, before any orthodontic treatment and alveolar bone grafting. The following were our exclusion criteria: the subjects with bilateral CLP, cleft lip and alveolus and isolated cleft palate, Syndromic UCLP, cheiloplasty and palatoplasty had not been performed. Following the strict inclusion and exclusion criteria, all subjects were selected by simple random sampling from the record archive of the hospital with proper permission from the authority. This study included 84 dental models of non-syndromic UCLP children. The age range of the individuals was 5–12 years where the average range was 7.69 (SD 2.46) years. Among the selected subjects, 43 subjects were male and 41 were female. Fifty subjects had a family history of cleft and 34 subjects had a family history of skeletal class III malocclusion (mandibular prognathism and/or maxillary retrognathism). Among them 18 subjects both had a family history of cleft and class III malocclusions. Thirty-three patients had right-sided UCLP. Complete UCLP involves both hard tissue and soft tissue structures of the soft palate, hard palate, alveolus, lip and floor of the nostril. An incomplete UCLP does not involve the floor of the nostril. In this study, 53 subjects and 31 subjects had incomplete and complete UCLP. All subjects had undergone cheiloplasty at an average age of 5 months. In 35 subjects, the Milliard technique for lip closure had been performed, and in 49 subjects, a modified Milliard technique had been performed. All subjects underwent palatoplasty at an average age of 18 months. Forty-four subjects underwent the Bardach technique of palatoplasty, and 40 subjects underwent the V-Y pushback technique. In this study, all cheiloplasties and palatoplasties were performed at the same hospital, and two different surgeons performed all of the cases utilizing same treatment protocol for two different surgical techniques. Therefore, we could evaluate the techniques of surgery that had a poor effect on the DAR and PM. Both surgeons are well trained, highly skilled and specialists in this field having more than 20 years of experience in cleft surgery at that hospital.

### Sample size calculation

To study the prevalence of successful treatment outcomes using the EUROCRAN index,

**n =** ﴾**Z/∆)** ^**2**^ **×** P **(**1-P**)**.

whereZ = 1.96 (level of significance = 0.05); absolute precision, ∆ = 0.10 (10%); and anticipated population proportion, *P* = 0.317.

If the absolute precision is 10%, the sample size required is 84. (Table 1).

**Table 1:** Sample size calculation

Width of	
Δ	N
0.40/0.16	
0.35/0.1225	
0.30/0.09	9
0.20/0.04	21
0.10/0.01	84

For logistic regression analysis, the sample size is estimated by using a ratio 1 predictor: 12 cases. In our study, there are seven predictors. Therefore, 84 cases are required.

The DAR and PM were scored by the EI, which is a scoring system for the early, late-mixed and early-permanent dentition using four categories of antero-posterior, transverse and vertical discrepancies as well as three categories of PM in patients with UCLP [7]. In the case of the DAR, a score of 1 or 2 implies a favorable dental relationship, a score of 3 means a less-favorable antero-posterior or end-to-end relationship, and a score of 4 indicates that the patient will possibly require orthognathic surgery to correct the antero-posterior relationship. Similarly, in the case of PM, a score of 1 indicates good morphology, whereas a score of 3 indicates poor morphology [7]. Five calibrated examiners rated all 84 dental models twice at two-week intervals. Taking the data in each model together, we subsequently generated a mean score [22]. The subjects were divided into two groups: favorable (category ratings 1 and 2) and unfavorable (category ratings 3 and 4) for DAR. This grouping was used because patients in the favorable groups could be treated with conventional orthodontics, whereas patients in the unfavorable groups sometimes required surgical correction [23].

### Statistical analysis

The intra- and inter-examiner agreements were analyzed with kappa statistics. The Kappa values of the intra- and inter-examiner agreements were interpreted based on Altman [24]; where less than 0.20 indicated poor level of agreement; 0.21 to 0.40, a fair level of agreement; 0.41 to 0.60, a moderate level of agreement; 0.61 to 0.80, good agreement; and 0.81 to 1.00, very good agreement. Various factors with favorable and unfavorable outcomes were evaluated by the chi square test. Logistic regression analysis was performed using the dichotomous dependent variables of favorable and unfavorable groups. Both crude and backward stepwise logistic regression analyses were conducted to explore the unfavorable DAR in UCLP patients. These analyses were carried out using the statistical package SPSS Version 22.0 (SPSS Inc., Chicago, IL, USA). The significance level was set at *p* < 0.05.

## Results

### Reliability of EI

Intra-examiner agreements for examiners A, B, C, D and E were 0.881, 0.895, 0.911, 0.930 and 0.930,respectively, for DAR and 0.889, 0.892, 0.876, 0.915 and 0.935, respectively, for PM and showed very good intra-examiner agreements (Table 2). Kappa scores of inter-examiner agreements ranged from 0.0790 to 0.965 for DAR **(**Table 3 **)** and 0.725 to 0.891 for PM (Table 4 **)**. The kappa scores for the EI showed good to very good intra- and inter-examiner agreements.

**Table 2:** Intra-examiner agreements (Kappa statistics)

Intra-examiner agreements	Kappa value	Standard error
Dental arch relationship		
A	0.881	0.042
B	0.895	0.041
C	0.911	0.038
D	0.930	0.034
E	0.930	0.034
Palatal morphology		
A	0.889	0.048
B	0.892	0.047
C	0.876	0.049
D	0.915	0.041
E	0.935	0.037

**Table 3:** Inter-examiner agreements (Dental arch relationship)

Inter-examiner agreements	Kappa value	Standard error
First rating		
A vs. B	0.828	0.050
B vs. C	0.790	0.054
C vs. D	0.877	0.044
D vs. E	0.965	0.024
E vs. A	0.880	0.043
Second rating		
A vs. B	0.878	0.044
B vs. C	0.947	0.030
C vs. D	0.930	0.034
D vs. E	0.965	0.024
E vs. A	0.930	0.034

**Table 4:** Inter-examiner agreements (Palatal morphology)

Inter-examiner agreements	Kappa value	Standard error
First rating		
A vs. B	0.829	0.058
B vs. C	0.725	0.071
C vs. D	0.850	0.054
D vs. E	0.891	0.047
E vs. A	0.848	0.056
Second rating		
A vs. B	0.865	0.053
B vs. C	0.769	0.065
C vs. D	0.877	0.049
D vs. E	0.873	0.050
E vs. A	0.889	0.048

### Score distribution

**Fig. 1:**
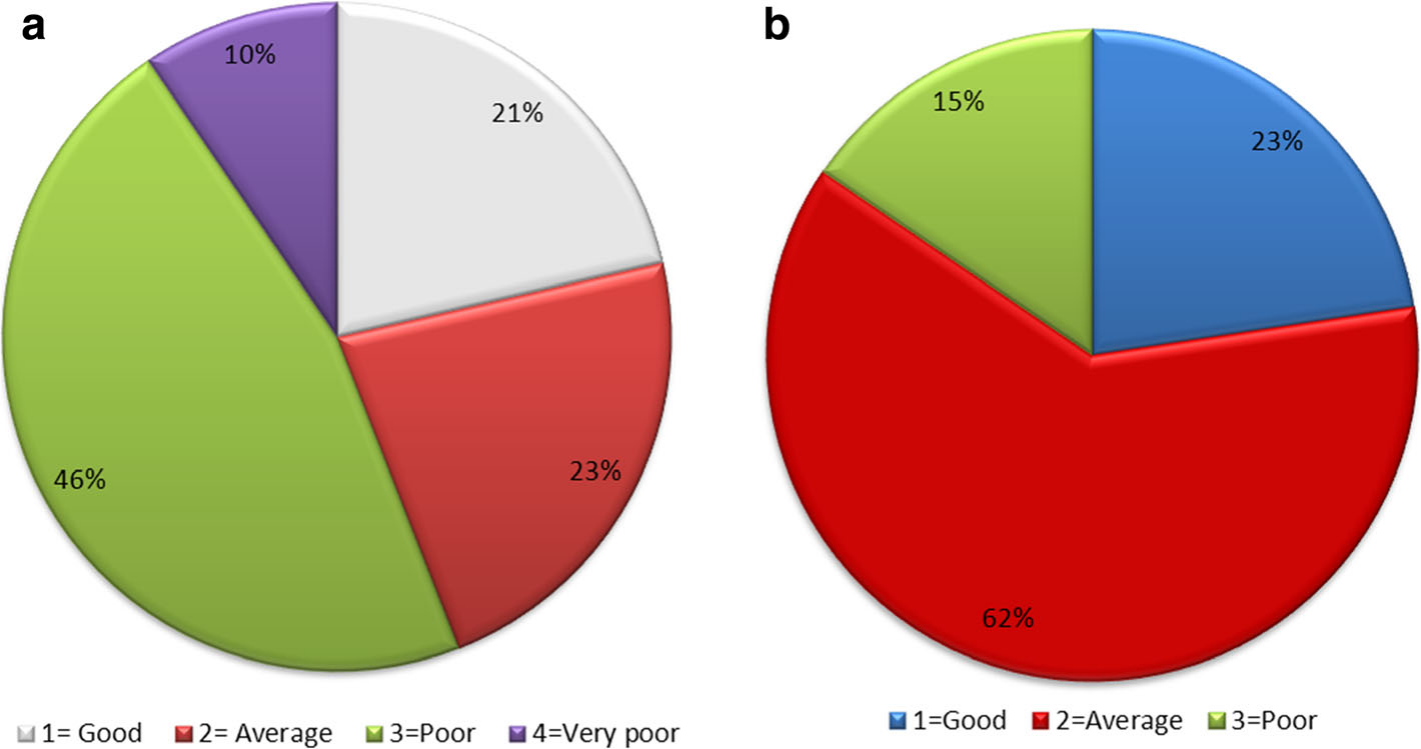
Score distribution of the EUROCRAN index for DAR (1**a**) and PM (1**b**)

Among the 84 subjects, the EUROCRAN scores were distributed as follows: category 1 = 18 subjects, 2 = 19 subjects, 3 = 39 subjects and 4 = 8 subjects for DAR (Fig. 1a),and category 1 = 19 subjects, 2 = 52 subjects and 3 = 13 subjects for PM (Fig. 1b).

### Comparison of factors between favorable and unfavorable groups

Table 5 shows the results of the distribution of the different factors, such as gender, UCLP type and side, family history of cleft and class III malocclusion, type of cheiloplasty, and type of palatoplasty, into favorable and unfavorable groups for DAR.

**Table 5:** Distribution of subjects with variable factors in favorable and unfavorable groups (the numbers of subjects in favorable and unfavorable groups were 37 and 47, respectively)

Variables	Favorable, n (%)	Unfavorable, n (%)
Gender		
Male	18(48.6)	25(53.2)
Female	26(63.4)	15(36.6)
UCLP affected side		
Left	20(54.1)	31(66.0)
Right	17(45.9)	16(34.0)
UCLP type		
Complete	10(27.0)	21(44.7)
Incomplete	27(73.0)	27(55.3)
Family history of cleft		
Positive	23(62.2)	27(57.4)
Negative	14(37.8)	20(42.6)
Family history of Class III		
Positive	16(43.2)	18(38.3)
Negative	21(56.8)	29(61.7)
Palatoplasty		
Bardach technique	21(56.8)	23(48.9)
V-Y pushback technique	16(43.2)	24(51.1)
Cheiloplasty		
Millard technique	18(48.6)	17(36.2)
Modified Millard technique	19(51.4)	30(63.8)

### Crude logistic regression analysis

In this study, a crude logistic regression analysis was performed to investigate the association between each factor and the DAR. The 95% confidence intervals were determined, and the factors with a *p*-value of less than 0.05 were considered to have a significant association with the DAR. Therefore, it can be concluded that the modified Milliard technique of cheiloplasty (*p* value 0.047) showed a significant association with unfavorable DAR (Table 6). In addition, complete UCLP (odds ratio 2.921) appears to be responsible for unfavorable DAR because its odds ratio is higher (>1) (Table 6).

**Table 6:** Crude logistic regression analysis: Favorable vs. unfavorable group using EI (​* *P* < 0.05.)

Variable	Odds ratio	95% confidence interval	*P* Value
Gender (male)	1.034	0.397–2.691	0.945
UCLP affected side (left)	1.852	0.653–5.255	0.247
UCLP type (incomplete)	2.921	0.613–13.916	0.178
Family history of cleft (+ ve)	0.554	0.187–1.643	0.287
Family history of Class III (+ ve)	0.931	0.344–2.516	0.888
Palatoplasty with Bardach technique	0.326	0.041–2.592	0.289
Cheiloplasty with Milliard technique	0.180	0.033–0.975	***0.047*** *

### Stepwise logistic regression analysis

A stepwise logistic regression analysis was performed to explore the association between various factors (independent variables) and the DAR (dependent variable). The significance level was set as <0.05. The regression analysis demonstrated that complete UCLP (*p* value 0.017) and the modified Milliard technique of cheiloplasty (*p* value 0.034) had a statistically significant influence on unfavorable DAR (Table 7).

**Table 7:** Stepwise logistic regression analysis (adjusted odds ratio; backward method): Favorable vs. unfavorable groups using EI (* *P*﻿ < 0.05.)

Variable	Odds ratio	95% confidence interval	*P* Value
UCLP type (incomplete)	4.142	1.285–13.351	***0.017*** *
Cheiloplasty with Milliard technique	0.296	0.096–0.914	***0.034*** *

### Rating of palatal morphology

**Fig. 2:**
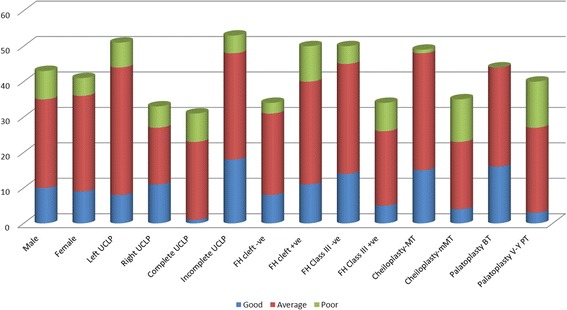
Rating of palatal morphology. CUCLP- Complete UCLP. ICULP-Incomplete UCLP. FH cleft +ve- Positive family history of cleft. FH cleft -ve- Negative family history of cleft. FH Class III + ve- Positive family history of Class III malocclusion. FH Class III -ve- Negative family history of Class III malocclusion. Cheiloplasty-MMT- Modified Millard technique of cheiloplasty. Cheiloplasty-MT- Millard technique of cheiloplasty. Palatoplasty BT- Bardach technique of palatoplasty. Palatoplasty V-Y PT- V-Y pushback technique of palatoplasty

Figure 2 shows the distribution of the different factors, such as gender, UCLP type and side, family history of cleft and class III malocclusion, type of cheiloplasty, and type of palatoplasty, that affect PM.

## Discussion

CLP patients face multitude of problems, both functional (sucking, deglutition, speech, ear, dental) and aesthetic, which is a lifelong burdening not only for patients but also for their parents [3]. Treatment outcomes for UCLP patients can be evaluated by various indices, such as the GOSLON Yardstick [12], the 5-year-old index [10], the GOAL index [25],the EUROCRAN index [7],the Huddart Bodenham scoring system [26],and the modified Huddart Bodenham scoring system [13, 14]. Among these, the EI is a relatively new, novel and moderately innovative tool for assessing the UCLP patient and has been shown to have moderate to very good inter- and intra-examiner reliability [7]. Unlike many index, the EI can evaluate not only surgical outcomes but also the degree of malocclusion in both antero-posterior and vertical aspect, as well as the palatal outward appearance [27]. It should be noted, however, that other indices, such as the GOSLON Yardstick [12] and the modified Huddart Bodenham for crossbite [13, 14], can evaluate only the DAR.

In our study, we evaluated treatment outcomes of the DAR and PM of Bangladeshi UCLP children to explore the responsible congenital/phenotype and postnatal treatment factors that affect treatment outcomes. Therefore, 84 dental casts were taken of non-syndromic UCLP patients before orthodontic treatment and alveolar bone grafting. Patients ranged in age from 5 to 12 years. All of the dental casts were assessed using the EI. The DAR of the EI showed very good intra- and inter-examiner agreement (Tables 2 and 3). Similarly, intra- and inter-examiner agreement of the PM was good (Tables 2 and 4). However, the treatment outcome of the DAR was poor to very poor, representing 56% of the cases. Of the remaining cases, 23% were average and 21% had a good prognosis (Fig. 1). Moreover, 62% of subjects demonstrated moderate outcomes, whereas 23% had good and 15% had poor outcomes of PM (Fig. 1).

Only two studies had been published to date that used the EI [7, 11]. Using the EI, Fudalej et al. [7] compared DARs based on two types of palatoplasty (one-stage and three-stage) between two different groups of people and found that one-stage palatoplasty was responsible for poor DAR. Conversely, one-stage palatoplasty was favorable for PM. In another study, Fudalej et al. [11] compared the outcome of the DAR between two groups of palatoplasty (exposed and unexposed) using the same index and found that the unexposed group had favorable DAR outcome, though no effect was found on PM. In our study, we evaluated the DAR based on various congenital/phenotype factors (gender, UCLP type, UCLP side, family history of cleft and family history of class III malocclusion) and postnatal treatment factors (cheiloplasty and palatoplasty) that are responsible for poor DAR and PM. Table 8 shows the mean score of DAR and PM globally and in the present studies using the EI.

**Table 8:** Mean score of DAR and PM globallyand in the present studies using the EI

Author	Number of sample (n)	Mean score (SD)	
		Dental arch relationship	Palatal morphology
Fudalej et al. (2011) [7]	Warsaw group-61	2.58 (0.92)	1.79 (0.43)
	Nijmegen-97	1.97 (0.88)	1.96 (0.55)
Fudalej et al. (2012) [11]	Exposed group-47	3.04 (1.00)	1.88 (0.57)
	Unexposed group-61	2.63 (0.97)	1.81 (0.55)
Present study	Male-43	2.51 (0.95)	1.95 (0.65)
	Female-41	2.37 (0.94)	1.90 (0.59)
	Left UCLP-51	2.53 (0.86)	1.98 (0.55)
	Right UCLP-33	2.30 (1.05)	1.85 (0.71)
	CUCLP-31	2.74 (0.85)	2.23 (0.50)
	IUCLP-53	2.26 (0.94)	1.75 (0.62)
	FH cleft +ve-50	2.42 (0.95)	1.98 (0.65)
	FH cleft -ve-34	2.47 (0.93)	1.85 (0.56)
	FH class III + ve-34	2.47 (0.86)	2.08 (0.62)
	FH class III -ve-50	2.42 (0.99)	1.82 (0.60)
	Cheiloplasty-MT- 35	2.40 (0.95)	2.23 (0.65)
	Cheiloplasty-MMT- 49	2.47 (0.94)	1.71 (0.50)
	Palatoplasty BT- 44	2.32 (0.96)	1.63 (0.49)
	Palatoplasty V-Y PT-40	2.58 (0.90)	2.25 (0.59)

To explore the associations between each congenital/phenotype and postnatal treatment factor and the DAR, a crude logistic regression analysis was carried out. A stepwise logistic regression analysis was used to explore the responsible factors and DAR. Using crude and stepwise backward regression analyses, significant associations were found between the modified Millard technique of cheiloplasty and unfavorable DAR (*p* value 0.047 and 0.034, respectively). Complete UCLP (*p* value 0.017) was also significantly associated with unfavorable DAR. In addition, the chi square test showed no significant differences found between the favorable and unfavorable groups of DAR, but type of UCLP, type of cheiloplasty and type of palatoplasty showed significant associations with PM.

Regarding cheiloplasty, several surgical protocols have been used, such as the Tennison technique, Millard technique, modified Millard technique, Olekas technique, and Randall technique [28], and researchers have evaluated the outcomes. In our study, we evaluated the Millard and modified Millard techniques for cheiloplasty and our results showed unfavorable outcome with the modified Millard technique for cheiloplasty. Others authors [16, 20, 21, 29] have also found significant differences. Kajii et al. [20] and Alam et al. [21] evaluated Japanese patients with UCLP and found that the modified Millard technique with vomer flap had a poor effect on DAR; this study used a different index. However, in another study, the Millard technique showed tremendous results with narrow clefts, whereas the Tennison technique showed more flexibility with wide clefts [29]. For both esthetic and functional purpose, the Onizuka technique provided high satisfaction not only to the patients but also to the surgeons [16].

For palate repair in UCLP subjects, Von langen beck’s technique, the Bardach technique, the V-Y pushback technique, one-stage palatoplasty, two-stage palatoplasty, three-stage palatoplasty, and Furlow’s technique are usually performed [30]. However, in Bangladesh, the Bardach technique and V-Y pushback technique are commonly used to repair the palate. Both methods involve palatoplasty with exposed surface types. Our results showed that the Bardach technique leads to favorable results without any significant differences for DAR and PM. In a previous study, Alam et al. [21] found the pushback technique to be unfavorable. However, Liao et al. [31] found that the two-flap technique had a better effect on maxillary growth than the vomar flap technique, whereas another study revealed that the two-stage closure with delayed repair technique shows comparatively better maxilla growth [32]. A future study of the Bangladeshi population using other techniques of palatoplasty without exposed surface should try to evaluate treatment outcomes.

Weighing the of all evidence, it should be stated that this study provides new information that both phenotype factors (complete UCLP) and postnatal treatment factors (modified Millard technique of cheiloplasty) are responsible for unfavorable DAR and PM in Bangladeshi UCLP children. These findings were obtained by assessing Bangladeshi UCLP children using the EI, but may be different in other populations. Therefore, we encourage other countries to conduct similar studies.

## Conclusion

The present study shows the mean scores of the EI for the DAR and PM were 2.44 and 1.93, respectively. As well as, the complete type of UCLP and the modified Millard techniques of cheiloplasty had a significantly unfavorable effect on both the DAR and PM in UCLP children.

## Abbreviations

CLP: Cleft lip and palate
DAR: Dental arch relationship
EI: EUROCRAN index
PM: Palatal morphology
UCLP: Unilateral cleft lip and palate
